# Is an in-home telerehabilitation program for people with proximal humerus fracture as effective as a conventional face-to face rehabilitation program? A study protocol for a noninferiority randomized clinical trial

**DOI:** 10.1186/s13102-016-0051-z

**Published:** 2016-08-26

**Authors:** François Cabana, Catherine Pagé, Amy Svotelis, Samuel Langlois-Michaud, Michel Tousignant

**Affiliations:** 1 Department of surgery, Faculty of Medicine and Health Sciences, Université de Sherbrooke, 3001 12e Avenue Nord, Sherbrooke, J1H 5N4 QC Canada; 2 Research Centre on Aging, Centre intégré universitaire de santé et de services sociaux de l’Estrie - Centre hospitalier universitaire de Sherbrooke (CIUSSS de l’Estrie CHUS), 1036 Belvédère Sud, Sherbrooke, J1H 4C4 QC Canada

**Keywords:** Rehabilitation, Proximal humerus fracture, Telerehabilitation, Effectiveness

## Abstract

**Background:**

Proximal humerus fractures can be treated surgically (eg: pinning, plate and screws) or conservatively by wearing a splint or a cast. Following both of these approaches, rehabilitation has proven effective to prevent functional limitations and to re-establish normal shoulder function. However, access to these rehabilitation services and compliance tends to be limited in elderly patients due to travelling difficulties caused by their precarious health status and, in some cases, social and marital status. Since the majority of patients with a proximal humerus fracture are elderly, it becomes relevant to find a new way to offer quick, simple and suitable rehabilitation service. Thus, the use of promising alternative approaches, as in-home telerehabilitation, can enhance access to rehabilitation services for such population. The main objective of the study is to compare the clinical effects of the innovative telerehabilitation approach (TELE group) compared to face-to-face visits to a clinic (CLINIC group) for patients treated for a proximal humerus fracture.

**Methods/Design:**

In this randomized controlled trial, individuals who have had a proximal humerus fracture treated conservatively at the Centre intégré universitaire de santé et de services sociaux de l’Estrie - Centre hospitalier universitaire de Sherbrooke (CIUSSS de l’Estrie CHUS), and who are returning home will be included. Participants will be recruited during their visit to the emergency ward or outpatient clinic by the medical or research team and will then sign the informed consent form if they are interested to participate in the study. We expect to recruit 52 participants (26 per group). Randomization will be done by a random number generator with sealed envelopes. Each patient will be evaluated before the beginning of the rehabilitation (T1), and immediately after the 2-month intervention (T2). The following outcomes will be measured: 1) upper extremity function (Constant Shoulder Score and Disability of the Arm, Shoulder and Hand questionnaire [DASH]); 2) range of motion (conventional goniometer); 3) user satisfaction (Health Care Satisfaction questionnaire); and 4) cost of services to the public healthcare system.

The difference between the two groups will be compared using a *t*-test or a chi-squared test, and through a cost-effectiveness economic analysis.

**Discussion:**

We hypothesize that in-home telerehabilitation will provide a good alternative to conventional rehabilitation, in terms of its efficacy, simplicity, patient satisfaction, and low associated costs.

**Trial registration:**

ClinicalTrials.gov: NCT02425267. April 22^nd^, 2015.

## Background

The aging population has placed in interesting pressure on our health system. In addition to different age-related conditions, the prevention and treatment of fragility fractures has become an important issue. Although osteoporosis treatments have improved in recent years, in addition to falls, the associated fractures are highly related to morbidity and mortality. Each year, one person out of three falls, and from them, 10 % causes a fracture [1]. The proximal humerus is one of the three most common fractured sites, along with the hip and wrist, making up 4 to 5 % of fractures, all sites combined [2].

Humerus fractures can be treated surgically (e.g. pinning, plate and screws, etc), or conservatively, typically through immobilisation in a splint or an orthotic device [3]. Conservative medical treatment tends to be advocated, mostly because of the complications that may occur following surgical intervention. However, the type of fracture, the surgeon, the patient and the fracture classification will influence the treatment decision. Among the existing proximal humerus fracture classifications (i.e. Neer [4], AO [5], Codman-Hertel [6], and Resch [7]), the Neer classification is the most widely used among orthopaedists. Neer group I and II fractures are often treated with the conservative approach, while group III and IV fractures most likely are surgical cases [8, 9]. Following both of these medical approaches, the individual needs rehabilitation to prevent functional limitations. Indeed, intervention programs in rehabilitation for humerus fractures have demonstrated their efficacy to improve shoulder joint mobility, diminish pain and improve functional state [10, 11].

Immobilisation time between the fracture and the beginning of rehabilitation is a parameter that has a significant influence on conservative treatment outcome [10–12], and, in reality, varies greatly between cases. A recent randomised clinical trial (RCT) [12] addressed the question of the efficacy of an immediate versus delayed mobilisation, showing that patients with a shorter immobilisation time (1 week) recovered faster functionally than patients with a longer immobilisation time. A similar study demonstrated that, in addition to a faster functional recovery, an immediate rehabilitation following the fracture leads to experiencing less pain for the patient [11]. Another study even claimed that a 3-day immobilisation, followed by passive mobilisation is sufficient and safe to restore physical capacities and post-fracture performance [10]. Finally, a RCT has compared the difference between the efficacy of a conventional physical therapy treatment and an individual in-home training program with instructions [13]. The results demonstrated no significant difference between treatment types. Interestingly, advantages emerged in the individual approach with instructions, such as: 1) allowing the patient to stay at home while receiving their rehabilitation, eliminating the need to travel to a clinic, and 2) patient satisfaction derived from having the responsibility of their rehabilitation at home.

Presently, in Canada, rehabilitation for proximal humerus fractures occurs at the hospital (37.2 %) [14], in external clinics, and in various in-home services. However, not all patients have access to rehabilitation services, mainly due the difficulty of the elderly to travel to the clinic because of their precarious health condition [15]. Therefore, new service delivery strategies are essential to enhance accessibility to rehabilitation services, with a focus on in-home services. Telerehabilitation, defined as a telehealth application that uses telecommunication technologies to provide physical therapy services [16–19], is an interesting solution to the lack of available services. This approach allows the patient to receive rehabilitation at home, without the need for a health care professional to travel to the patient’s home or to travel to the clinic for the patient. As such, telerehabilitation could permit the elderly with a proximal humerus fracture to have access to rehabilitation directly in their home with adequate and safe supervision. Services provided by telerehabiliation can be very diverse, including telecheckups (phone calls to ensure the wellness of the patient), telemonitoring (record physiological data), teleconsultation (between two service centers) and teletreatment [19, 20]. This long-distance therapeutic intervention also includes the notion of frequency (a set number of sessions per week) and duration (over a specific time period) [21, 22].

A systematic review of telerehabilitation interventions [18] showed a tendency for these services to generate clinical improvements that are generally equal to those resulting from conventional rehabilitation programs. For example, efficacy of teletreatment has been demonstrated: in older populations with loss of autonomy [23], or living at home will mild to moderate dementia [24]; in individuals with mobility impairments [25]; and following total knee [26–29] or shoulder [30] arthroplasty. Interestingly, in the latest study [30], rehabilitation treatment was provided to 10 elderly patients through a videoconferencing system installed directly in their home. The intervention period length was 2 months and included supervised sessions, where a physical therapist was able to adapt exercises according to each patient’s evolution. Results of this study demonstrated that patients are able to adhere to teletreatment, and that they are able to maintain a good relationship with their physical therapists even though there was no direct physical interaction. Moreover, this remote method of receiving their rehabilitation facilitated their daily living since they were not required to travel to receive treatment. By staying home, patient motivation increased and they felt more independent in their exercises. However, an important limitation to this study was the lack of evaluation of clinical results. Therefore, although a promising avenue for rehabilitation, especially in older populations, the efficacy and functional results of telerehabilitation remain to be studied with high quality evaluations.

To our knowledge, only one study exists on telerehabilitation among patients with a proximal humerus fracture [31]. This study, realised by our research team, demonstrated the feasibility of such a service delivery to this specific population. However, our previous study was more focussed on proof-of-principle, and as such, the lack of a control group (treatment at clinic) did not permit us to affirm that clinical improvement was due to the patient’s natural recovery or to the telerehabilitation received. Thus, the main objective of the present study is to evaluate the efficacy of in-home telerehabilitation (TELE group) compared to the conventional rehabilitation in a clinic (CLINIC group) in a population with a proximal humerus fracture. Our hypothesis is that in-home telerehabilitation will prove to be a good alternative to conventional rehabilitation.

## Methods/Design

### Study design

**Fig. 1:**
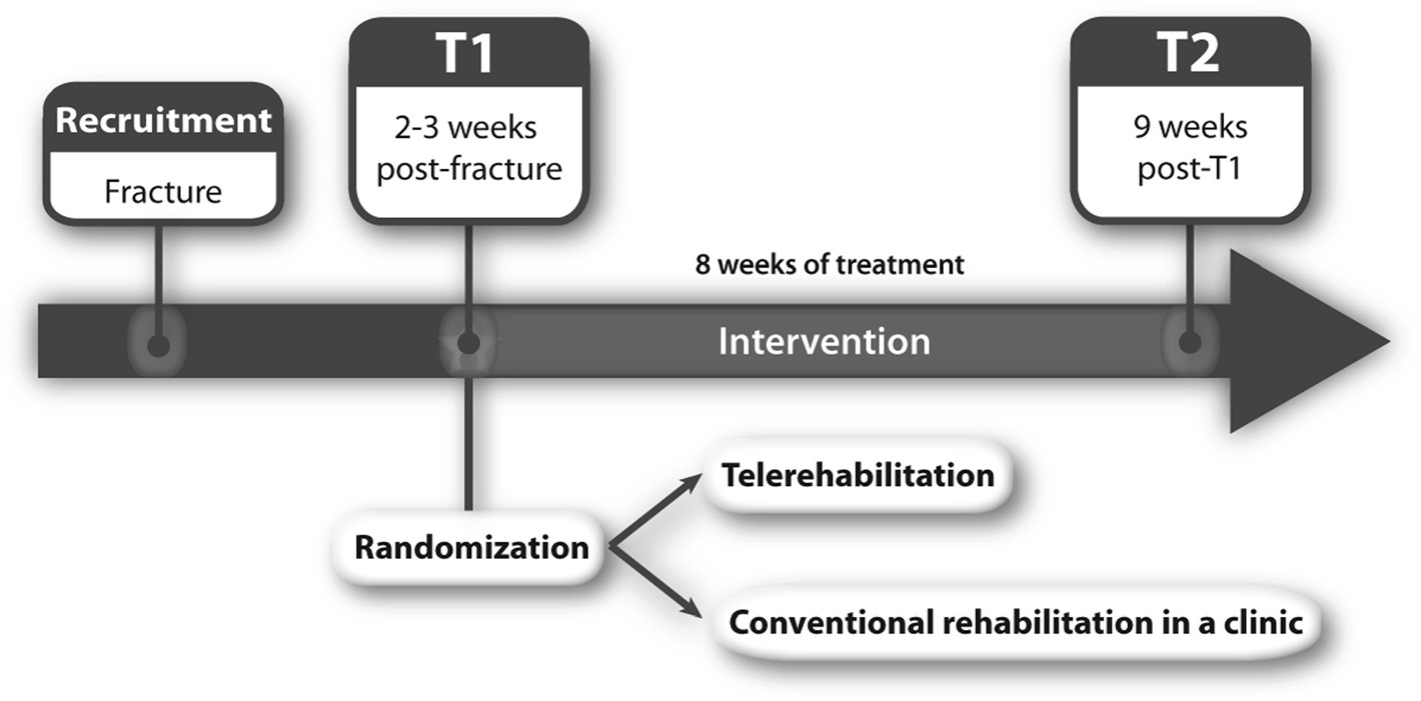
Study timeline. The patient is recruited post-fracture and evaluated at baseline (T1). Then, the participant is randomized into either Telerehabilitation group or Conventional rehabilitation group. Following 8 weeks of treatments, the patient is evaluated again (T2)

In our study, teletreatment will be used to provide physical therapy sessions from a service center (clinic) directly into the patients’ home. The study design is a RCT. As described in Fig. 1, there will be an evaluation at baseline (T1), 1 to 2 weeks post-fracture. Then, the 8-week intervention phase (telerehabilitation) or conventional rehabilitation in a clinic will begin at the moment prescribed by the orthopaedist, approximately 2 to 3 weeks post-fracture. Finally, a second evaluation (T2) will be held at the end of the intervention period.

### Participants

This study will be conducted in the population with a proximal humerus fracture treated conservatively at the Centre intégré universitaire de santé et de services sociaux de l’Estrie - Centre hospitalier universitaire de Sherbrooke (CIUSSS de l’Estrie CHUS). To be included, participants will have to: 1) return home after discharge from hospital or emergency; 2) be apt to do exercises; 3) have a sufficient verbal and written comprehension to participate to the treatment and evaluations; 4) have access to high speed internet connection at home. Participants with the following characteristics will be excluded from the study: 1) intra-articular proximal humerus fracture types (often susceptible to longer rehabilitation periods and a higher risk of complications); 2) presence of any other upper-limb fracture that can interfere with rehabilitation; and 3) surgical treatment following the fracture.

A sample of 52 participants with a proximal humerus fracture and responding to the eligibility criteria will be included. Participants will be screened by the orthopaedic research team through the orthopaedic resident or nurse who proceeds to the installation of the removable splint. At that time, the research team will briefly inform the patient on the study and will obtain his/her authorisation to be contacted by phone by a member of the teletreatment research for a more detailed evaluation of eligibility and explanation of the study. Following this telephone interview, if the participant is still interested and meets all the eligibility criteria, an appointment will be scheduled to obtain the informed consent (approved by local ethic committee; CIUSSS de l’Estrie - CHUS, and perform the first evaluation (T1). Immediatly following visit T1, each patient will be randomised to either the TELE or the CLINIC group. The randomised list has been generated electronically using block randomization of size 4. The evaluator is the only one who will be blind to the randomisation.

### Independent variable: rehabilitation program

The rehabilitation program is identical in both randomised groups, and will be dispensed by physical therapists of the Clinique universitaire de réadaptation de l’Estrie (CURE). Only the delivery mode will differ; TELE group (in-home telerehabilitation) or CLINIC group (conventional face-to-face rehabilitation in a clinic). The intervention consists of a rehabilitation program with constant qualified physical therapist supervision. The exercise program, based on a post-prosthesis and post-fracture rehabilitation program developed by the orthopaedic surgery division of the CIUSSS de l’Estrie - CHUS, includes stretching, pain management, range of motion and muscular strengthening, in addition to a question period. An example of a rehabilitation session is described in Table 1. The attending physical therapist will also adjust the exercises according to the progression of each patient’s condition (see Table 2 for the rehabilitation session progression).

**Table 1:** Example of a rehabilitation session

Length (minutes)	Exercise types
≈5 à 10	Warm-up and stretching
≈15 à 30	According to progression • Weeks 1–2 : Pain management and range of motions • Weeks 3–4 : Range of motion renewal (active assisted) • Weeks 5 à 8 : Range of motion maintaining (active) and muscle strengthening
≈5 à 10	Question period
Total : ≈ 30–45	End of the intervention

**Table 2:** Rehabilitation session progression

Exercices	Weeks^a^							
	1	2	3	4	5	6	7	8
Circulatory movements	x	x						
Pendulum movements	x	x						
Wrist and elbow movements	x	x						
Thermal method (if necessary)	x	x	x	x	x	x	x	x
Range of motion exercices			x	x	x	x	x	x
Muscle strengthing					x	x	x	x

^a^Week 1 of rehabilitation matches to approximately week 3 post-fracture

The training program consisting of 30 to 45-min sessions, which will be realised for 8 weeks at a frequency of twice daily, either supervised (TELE or CLINIC) or unsupervised at home. During weeks 1, 3 and 5, patients will have to perform their exercises twice with direct supervision of the physiotherapist and others without this supervision. For the other weeks (2, 4, 6, 7, 8), patients will only have one supervised sessions, and the others without supervision. Supervised sessions will allow both the therapist and the patient to adjust the program if a problem occurs and assure the proper execution of the exercises.

#### Telerehabilitation technological platform

**Fig. 2:**
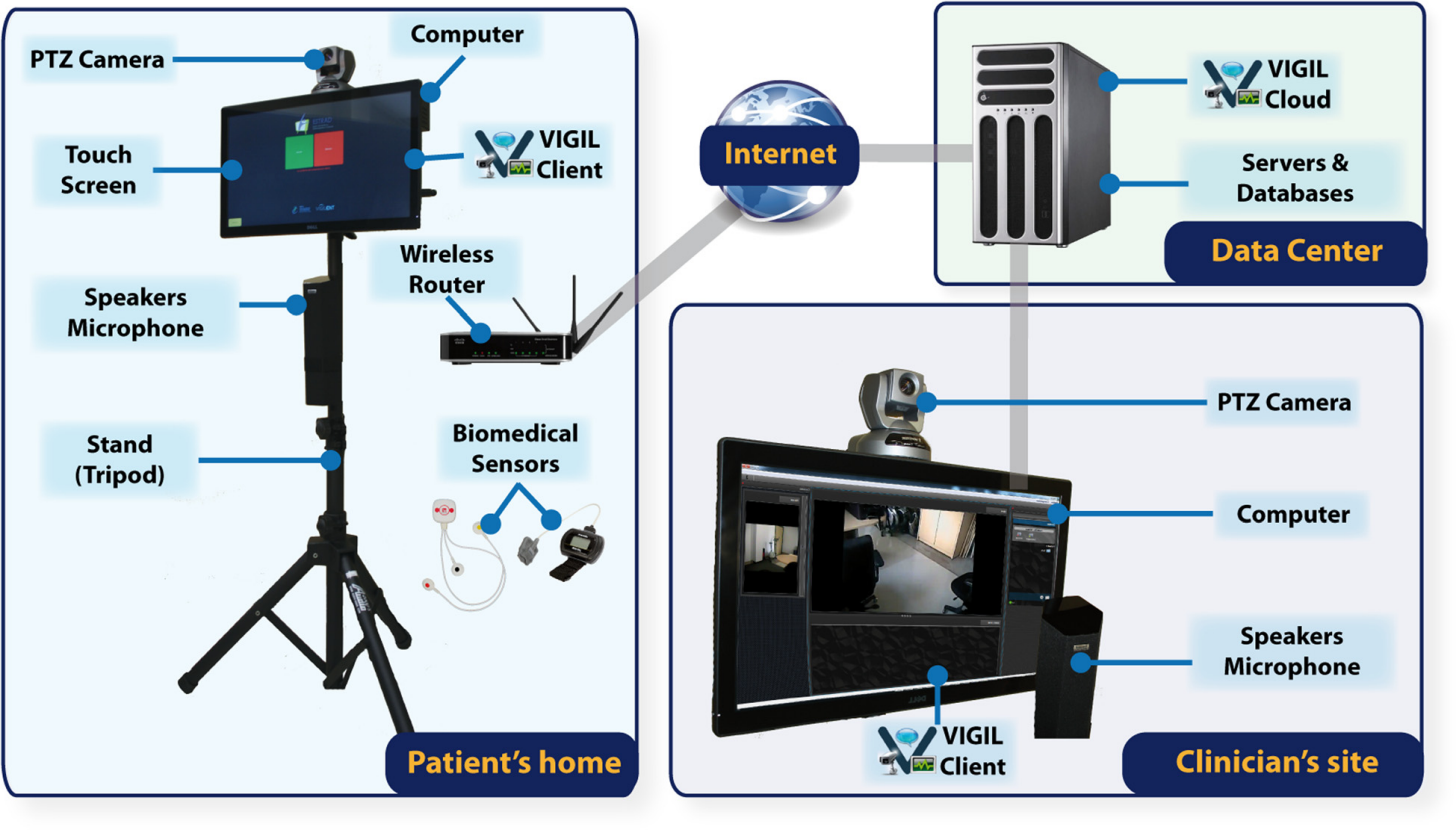
Telerehabilitation technological platform. The patient and clinician systems include a 22″ touch monitor, a mini-PC (Intel NUC), a pan-tilt-zoom (PTZ) camera with embedded h264 video codec, a microphone array and a speaker. The telerehabilitation software, Vigil2, runs on both systems. The software includes functionalities for management (users, systems and sessions), patient status (online, offline, previous sessions, planned sessions), secure video, audio and data transfer over the Internet, and intuitive camera control (point-and-click control scheme). It also includes an easy way for the patient to turn on and off the system using the touch screen. Audio, video and sensor data coming from the patient’s home are transferred to the clinician using an application and database server over a secure link, allowing real-time sessions to occur

The originality of the in-home telerehabilitation intervention stems from the superior level and type of interaction between users (health care professional and patient), which exceeds the usual use of a videoconferencing system. As such, the technological support must be flexible in order to respond to the needs and constraints of such a system. The telerehabilitation platform used in this study was developed in collaboration with Vigilent Telesystems to address these issues. Figure 2 describes the telerehabilitation platform used in the study.

### Dependent variables

Participants will attend a total of two evaluations of approximately 1.5 h each with a trained research assistant at the Research Centre on Aging before (T1) and after (T2) the intervention period. Each assessment will be executed in the same order to optimise the validity of the collected information: 1) range of motion (flexion, extension, abduction, external and internal rotation); 2) upper limb function; 3) global shoulder function and pain; and 4) participant satisfaction. Every item will be evaluated at both assessments, except for the satisfaction, which will be assessed only at T2.

#### Range of motion

Shoulder range of motion (flexion, extension, abduction, and internal and external rotations) will be assessed by a universal goniometer [32]. This standard, simple and reliable instrument used to measure angles has a degree-graduated scale. Active and passive measurements will be taken with standardised procedures.

#### Upper limb function

Shoulder function will be evaluated with the the Disability of the Arm, Shoulder and Hand questionnaire (DASH) [33]. This self-administered questionnaire includes 30 questions evaluated on a 5-point Likert scale, most of which relate to the individual’s capacity to realise a task. This tool was chosen for its scientific validity, ease of use, and ability to accurately reflect activity levels in daily living. The result is on a total of 100, where a high score indicates a greater disability. The original English version questionnaire demonstrated a good test-retest reliability (intraclass correlation coefficient = 0.95), a good internal consistency (Cronbach alpha = 0.96) and a moderate construct validity according to Spearman correlations varying from -0.58 to -0.76 [34]. However, factorial analyses demonstrated that five factors explained 67 % of total variance [35].

#### Shoulder functional measures

Shoulder global function will be measured with the Constant score [36], which is the primary outcome measure. This questionnaire allows the assessment of four outcomes related to shoulder function: 1) pain; 2) activities of daily living (sleeping, work, leisure); 3) range of motion; and 4) muscle strength. The total is on 100 and a higher score indicates a higher shoulder function. According to a systematic review of the psychometric properties of the Constant score, the reliability of this questionnaire is excellent (ICC, 0.89; 95 % confidence interval, 0.79–0.94) [37], and the internal consistency, evaluated with the Cronbach alpha, ranges from 0.60 to 0.75 suggesting that it measures different aspects of function [38, 39].

#### Patients’ satisfaction

Every patient will complete the French version of the validated Healthcare Satisfaction Questionnaire at T2 to evaluate their general satisfaction toward the health care service received [40]. This questionnaire also showed a good internal consistency (Cronbach’s alpha coefficient of the overall scale = 0.92) [40]. Satisfaction construct is determined by three distinct factors, whether satisfaction with the: 1) relationship with the healthcare professional; 2) services delivered; and 3) general healthcare organization. A score on 100 is computed for the general satisfaction, as well as for satisfaction of each domain.

In addition, patients’ satisfaction toward teletreatment (only for the TELE group) will be measured after removal of the technology by the technician using the Telemedicine Perception Questionnaire [41]. This validated questionnaire includes 14 items placed on a five-point Likert scale (where 1 = totally disagree and 5 = totally agree) for a total score of 56. The first six items explore the communication quality between the professional and the patient, and the other items concentrate on the patient’s perception of the quality of the received service, including coherence, accessibility and needs met.

#### Costs

Economic analyses are based on the health system perspective [42]. A grid previously developed and already used to collected costs associated to teletreatment (TELE group) and conventional rehabilitation (CLINIC group) for our post-knee arthroplasty [43] will also be part of this study.

### Statistical methods

The principal analyses intend to test the noninferiority of the TELE intervention versus the CLINIC intervention. We aim to recruit a total of 52 participants, or 26 participants per group. Sample size was determined with data from a previous study on the Constant score [44], which is considered in this study as the primary outcome measure. With 26 participants per group, within the context of a unilateral t-test with an alpha level set at 0.05, the power of the study to reject non-equivalence hypothesis would be at 69 % for a difference of 10.3 or less (standard deviation = 17). This noninferiority point is under the minimal clinically important difference of 10.4 points on the Constant score [44].

Participant characteristics in each group will be described pre-intervention (T1) using mean and standard deviation (continuous variables) or proportion (categorical variables). Groups at baseline will then be compared using *t*-test or chi-squared test.

First, the data will be analysed according to the received intervention (per protocol), and then, according to the assigned group (intention to treat analysis). A sensitivity analysis will be used to explore the effect of compliance or screen failures: missing data will be replaced by extreme data (no change following intervention or most favorable change noted in the study). Any non-robustness of the revealed results by comparison of strategies will be noted.

Economic analysis is of cost-efficacy type. Cost by change-unit of the principal dependant variable (Constant score) will be determined for both groups. Differential cost will then be established.

## Discussion

This trial is the next logical step to the feasibility study conducted by our research team on 12 post-proximal humerus facture patients [31]. The previous study demonstrated that telerehabilitation seems to be a promising avenue to provide rehabilitation services to this population without adverse events. The results obtained following the present protocol will affirm the cost-efficacy of telereahabilitation treatment in an orthopaedic context, more precisely, after a proximal humerus fracture treated conservatively.

In order to control a potential selection bias, randomisation will be blind to the evaluator and participants’ characteristics will be compared pre-intervention between each group. If a group differs on some characteristics despite randomisation, corrective measures will be made in subsequent analyses. Furthermore, information bias will be controlled by using standardized measures and by calibrating all the assessors for each assessment.

This study will verify the noninferiority of in-home telerehabilitation compared to face-to-face intervention at a clinic for patients with proximal humerus fracture. In accordance with our hypothesis, we think that telerehabilitation will improve access to a rapid, less expensive, satisfactory and effective rehabilitation services.

### Trial status

Recruitment has begun since June 2015.

## Abbreviations

CIUSSS de l’Estrie CHUS: Centre intégré universitaire de santé et de services sociaux de l’Estrie - Centre hospitalier universitaire de Sherbrooke
CLINIC: Conventional face-to-face rehabilitation in a clinic
CURE: Clinique universitaire de réadaptation de l’Estrie
DASH: Disability of the Arm, Shoulder and Hand questionnaire
RCT: Randomised clinical trial
T1: Baseline evaluation (pre-intervention)
T2: Post-intervention evaluation
TELE: In-home telerehabilitation
